# Supplementary material for: The interactome of intact mitochondria by cross-linking mass spectrometry provides evidence for coexisting respiratory supercomplexes
Source: Mol Cell Proteomics. 2017 Dec 8;17(2):216–32. doi: 10.1074/mcp.RA117.000470 (PMC5795388; doi:10.1074/mcp.RA117.000470)
Supplement: Supplemental Data [file supp_RA117.000470_133922_0_supp_23978_fzffwf.zip › spectra_annotation/mito_DR_spectra_annotation/273-1-4-1-13-1.pdf]

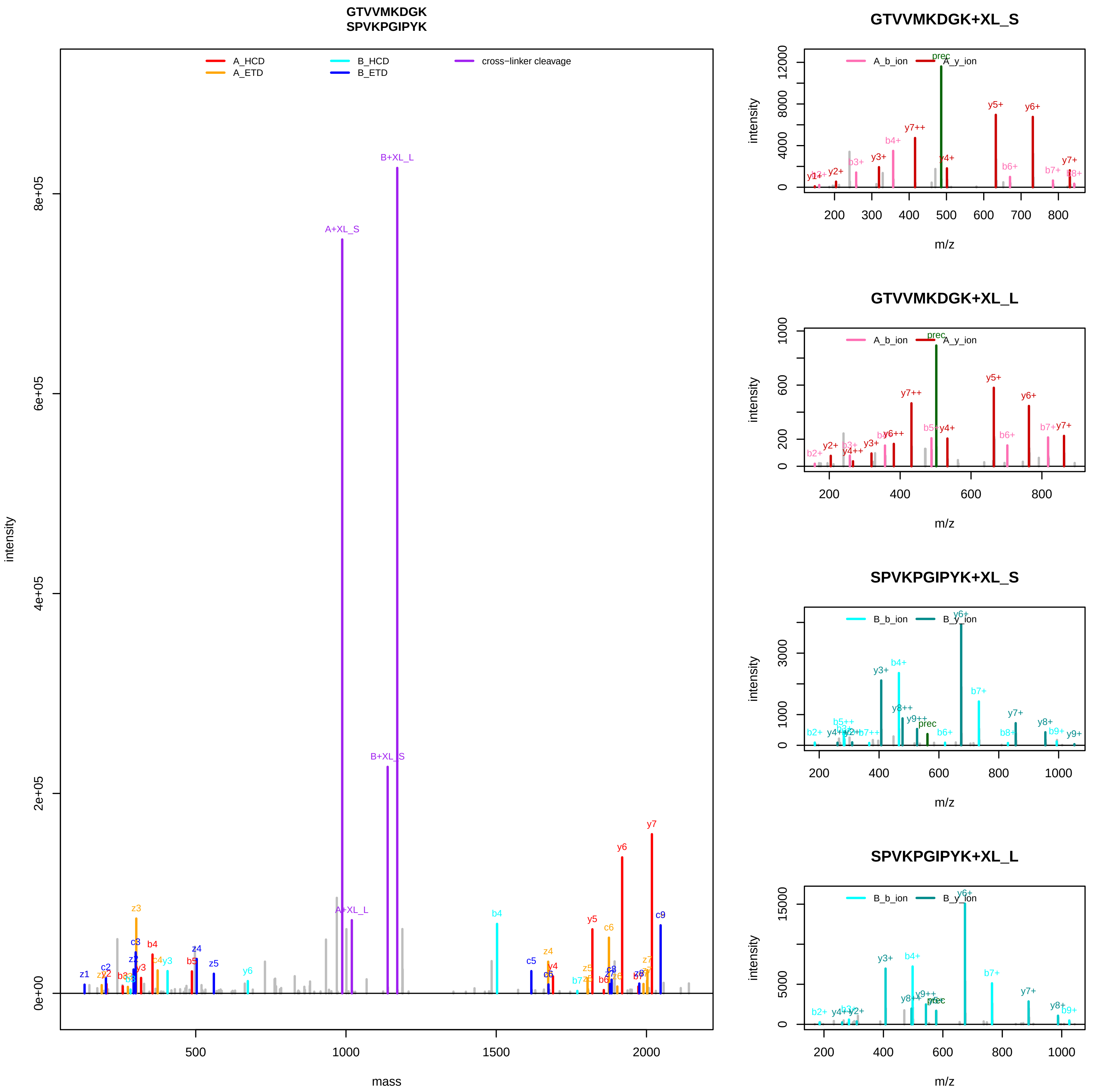

GTVVMKDGK
SPVKPGIPYK
A_HCD
A_ETD
B_HCD
B_ETD
cross−linker cleavage
B+XL_L
A+XL_S
B+XL_S
A+XL_L
intensity
mass
0e+00
2e+05
4e+05
6e+05
8e+05
500
1000
1500
2000
z1 c2 y2 b3 z3 c3 b4 c4 y3 b5 z4 z5 y6 b4 c5 z4 c6 y4 b7 z5 y5 b6 c6 y6 z8 b7 y7 c9
GTVVMKDGK+XL_S
A_b_ion
A_y_ion
prec
y5+ y6+ y7++ b4+ y3+ b3+ y2+ y4+ b6+ b7+ y7+ b8+ y1+ b2+
m/z
GTVVMKDGK+XL_L
A_b_ion
A_y_ion
prec
y5+ y7++ y6+ b7+ y7+ b5+ y4+ b6+ y6++ y3+ b3+ y2+ y4++ b2+
m/z
SPVKPGIPYK+XL_S
B_b_ion
B_y_ion
y6+ b4+ y3+ b7+ y8++ y9++ prec y7+ y8+ b9+ y9+ b2+ y4+ b5++ b3+ y2+ b7++ b6+ b8+
m/z
SPVKPGIPYK+XL_L
B_b_ion
B_y_ion
y6+ y3+ b4+ b7+ y8++ y9++ prec y7+ y8+ b9+ b2+ y4+ b3+ y2+
m/z
